# Abdominal migraine in children: association between gastric motility parameters and clinical characteristics

**DOI:** 10.1186/s12876-016-0435-2

**Published:** 2016-02-29

**Authors:** Niranga Manjuri Devanarayana, Shaman Rajindrajith, Marc A. Benninga

**Affiliations:** Department of Physiology, Faculty of Medicine, University of Kelaniya, Thalagolla Road, Ragama, 11010 Sri Lanka; Department of Paediatrics, Faculty of Medicine, University of Kelaniya, Thalagolla Road, Ragama, 11010 Sri Lanka; Department of Pediatric Gastroenterology and Nutrition, Emma Children’s Hospital, Academic Medical Centre, Amsterdam, The Netherlands

**Keywords:** Abdominal migraine, Abdominal pain, Functional gastrointestinal disorder, Gastric emptying, Gastrointestinal motility

## Abstract

**Background:**

Approximately 0.2–1 % of children suffers from abdominal migraine (AM). Pathophysiology of AM has not been adequately studied. This study evaluated gastric motility in children with AM.

**Methods:**

Seventeen children (6 boys), within an age range of 4–15 years, referred to a tertiary care paediatric unit, North Colombo Teaching Hospital Ragama, Sri Lanka, from 2007 to 2012, were screened. Those fulfilling Rome III criteria for AM were recruited after obtaining parental consent. None had clinical or laboratory evidence of organic disorders. Twenty healthy children (8 boys), with an age range of 4–14 years, were recruited as controls. Liquid gastric emptying rate (GE) and antral motility parameters were assessed using an ultrasound method.

**Results:**

Average GE (41.6 % vs. 66.2 %, in controls), amplitude of antral contractions (A) (57.9 % vs. 89.0 %) and antral motility index (MI) (5.0 vs. 8.3) were lower and fasting antral area (1.8 cm^2^ vs. 0.6 cm^2^) was higher in children with AM (*p* < 0.01). No significant difference in the frequency of antral contractions (F) (8.8/3 min vs. 9.3/3 min, *p* = 0.08) was found between the two groups. Scores obtained for severity of abdominal pain had a negative correlation with A (*r* = −0.55, *p* = 0.03). Average duration of abdominal pain episodes correlated with GE (*r* = −0.58, *p* = 0.02). Negative correlations were observed between duration of AM and A (*r* = −0.55), F (*r* = −0.52), and MI (*r* = −0.57) (*p* < 0.05).

**Conclusions:**

GE and antral motility parameters were significantly lower in children with AM. A significant correlation was found between symptoms and gastric motility. These findings suggest a possible role of abnormal gastric motility in the pathogenesis of AM.

## Background

Recurrent abdominal pain is a common symptom in children worldwide [1–5]. Majority of these children suffer from functional gastrointestinal disorders (FGIDs) [6–8] and only a minority have an identifiable organic cause [2, 7, 9, 10]. Previous studies have shown that approximately 10 to 12 % of children and adolescents suffer from abdominal pain predominant functional gastrointestinal disorders (AP-FGIDs) [11–13]. AP-FGIDs in children include irritable bowel syndrome (IBS), functional abdominal pain (FAP), abdominal migraine (AM) and functional dyspepsia (FD) [6–8].

AM is an uncommon AP-FGID in children. It is characterized by episodes of severe, intense periumbilical pain lasting for hours, associated with other intestinal and extra-intestinal symptoms such as headache, nausea, vomiting, photophobia and pallor [14]. Prevalence of AM varies from 0.2 to 4.1 % in community studies [11–13, 15, 16]. In hospital-based studies, AM is seen in 2.2 to 23 % of children with non-organic abdominal pain [7, 17–20].

Similar to other FGIDs, the exact underlying pathophysiology of AM is not clear [21, 22]. Various mechanisms, including gastrointestinal motility abnormalities, have been suggested as possible pathophysiological mechanisms for symptoms of FGIDs. Gastric motility abnormalities have been reported in children with other AP-FGID such as FD [23–25], IBS [26] and FAP [27]. However, there are no currently available data on gastric motility parameters in children with AM. In such a context, we attempted to study gastric emptying and antral motility parameters in children with AM and their correlation with symptoms.

## Methods

### Selection of patients with AM

This study was conducted in the Gastroenterology Research Laboratory, Faculty of Medicine, University of Kelaniya, Sri Lanka. All consecutive patients aged 4 to 15 years, referred to this laboratory from 1st January 2007 to 31st December 2012 and fulfilling the Rome III criteria for abdominal migraine (Table 1) [14], were recruited after obtaining consent of a parent. They were screened for organic diseases using detailed history and comprehensive physical examination (including growth parameters) and relevant investigations. Routine investigations done in all recruited patients to rule out organic disorders included stool microscopy, urine microscopy and culture, full blood count, C-reactive protein, liver and renal function tests. Special investigations performed in some patients based on clinical judgment included ultrasound scanning of the abdomen (*n =* 13), X-ray KUB (*n =* 2), serum amylase (*n = 5*), upper gastrointestinal endoscopy (*n = 2*) and lower gastrointestinal endoscopy (*n* = 1). None of the patients had evidence of organic disorders. The patients were followed up for a minimum of 3 months.

**Table 1:** Rome III criteria for abdominal migraine

H2c. Diagnostic Criteria^a^ for Abdominal Migraine
Must include *all* of the following:
1. Paroxysmal episodes of intense, acute periumbilical pain that lasts for 1 h or more
2. Intervening periods of usual health lasting weeks to months
3. The pain interferes with normal activities
4. The pain is associated with 2 or more of the following:
a. Anorexia
b. Nausea
c. Vomiting
d. Headache
e. Photophobia
f. Pallor
5. No evidence of an inflammatory, anatomic, metabolic, or neoplastic process considered that explains the subject’s symptoms

^a^Criteria fulfilled 2 or more times in the preceding 12 months

Exclusion criteria were clinical or laboratory evidence suggesting organic pathology, FGID other than AM, chronic medical or surgical diseases other than AM, children on long-term medications, previous abdominal surgery involving gastrointestinal tract, fever, common cold, respiratory tract symptoms, gastroenteritis or any other systemic infection during the previous month and subjects receiving drugs that can alter gastrointestinal motility during the previous month. List of drugs considered were benzamide, bethanechol, cinitapride, domperidone, erythromycin, itopride, levosulpiride, metoclopramide, mirtazapine, mitemcinal, mosapride, naloxone, prucalopride and renzapride. In addition, drug charts of all patients were reviewed to ensure that they had not taken any drugs that could change gastrointestinal motility during the previous month.

### Selection of controls

Healthy controls were selected from the same geographical area as the patients (Gampaha district of Sri Lanka). Total number of controls recruited was twenty. Their age ranged from 4 to 14 years. None of the controls had symptoms related to the gastrointestinal tract, such as abdominal pain, abdominal distension, constipation, diarrhoea etc. Written consent was obtained from a parent of all recruited controls.

### Assessment of symptom severity

All children with AM underwent gastric motility assessment during a period of abdominal pain. Severity of abdominal pain was graded as mild (1 – child is able to carry out regular activities during pain episodes), moderate (2 – child stops activities and sits down during pain episodes), severe (3 – child lies down during pain episodes) and very severe (4 – child cries or screams during pain episodes). This scoring system was adopted from Boey et al., [28]. It has been pretested for Sri Lankan children, and used in several previous Sri Lankan studies [1, 12].

### Assessment of relieving factors

The following 10 main relieving factors were addressed; massaging or pressing the painful area, applying ice or cold towels over the painful area, changing posture, becoming immobile during the attacks, isolating themselves, vomiting, defecation, sleep, eating or drinking and medications including drugs, home remedies and local applications.

### Assessment of exposure to stressful life events

We assessed exposure to 17 common school and family related stressful life events during previous three months and provided space to indicate any other event the patients were exposed to which in the child’s and parent’s view is stressful. The stressful life event questionnaire has been previously developed by the investigators, pretested and used in several previous Sri Lankan studies [1, 12, 29].

### Laboratory methods

For this study, gastric motility was assessed using a previously validated ultrasound method [30]. All ultrasound measurements were done by the same investigator (NMD). The investigator was not blinded for patients and controls.

All gastric motility measurements were done after an overnight fast, using a high-resolution, real-time scanner with a 3.5 MHz curve linear transducer. All subjects were examined seated in a chair, slightly leaning backwards.

The cross sectional area of antrum was measured in the fasting stage and after drinking a standard liquid meal heated to approximately 40 °C (200 mL of chicken soup, 54.8 kJ, 0.38 g protein, 0.25 g fat, 2.3 g sugar per serving, Ajinomoto Co., Tokyo, Japan). The meal was ingested within 2 minutes. The ultrasound probe was positioned vertically to permit simultaneous visualization of gastric antrum, superior mesenteric artery, abdominal aorta and the left lobe of the liver. The area of gastric antrum was measured by tracing the mucosal side of the wall using the built-in calliper and calculation program of the ultrasound apparatus. All measurements were done using the record and playback method.

Main gastric motility parameters assessed were fasting antral area, gastric emptying rate, frequency and amplitude of antral contractions and antral motility index.

#### Calculation of liquid gastric emptying rate

Maximum antral areas were calculated at 1st and 15th minutes after series measurements. Gastric emptying rate was calculated as the percentage reduction of gastric antral cross-sectional area at 15 min following ingestion of the liquid meal.

$$ \begin{array}{c}\hfill Gastric\  emptying\  rate\ \left(\%\right) = \Big[\left( antral\  area\  at\ 1 min\hbox{--} antral\  area\  at\ 15 mins\right)/\ \hfill \\ {}\hfill antral\  area\  at\ 1 min\Big]\mathrm{x}\ 100\hfill \end{array} $$

#### Calculation of antral motility

These antral motility parameters were calculated within the first 5 min after drinking the liquid meal. The minimum and maximum cross sectional areas of the antrum were measured during contractions and relaxations for at least 3 times to calculate the amplitude of antral contractions.

Antral motility parameters were calculated as follows:

$$ Frequency\  of\  antral\  contractions= Number\  of\  contractions\  per\ 3\  minute\  per iod $$

$$ \begin{array}{c}\hfill Amplitude\ \left(\%\right)=\Big[\left( antral\  area\  at\  relaxation\hbox{--} antral\  area\  at\  contraction\right)\hfill \\ {}\hfill / antral\  area\  at\  relaxation\Big]\mathrm{x}100\hfill \end{array} $$

$$ Motility\  index= Amplitude\  of\  antral\  contraction\ X\  Frequency\  of\  contraction $$

### Ethical approval

This study protocol was approved by the Ethics Review Committee, Faculty of Medicine, University of Kelaniya, Sri Lanka.

### Statistical methods

#### Calculation of sample size

Sample size calculation was done using WINPEPI statistical programme (Abramson, J.H. WINPEPI updated: computer programs for epidemiologists, and their teaching potential. Epidemiologic Perspectives & Innovations 2011, 8:1). Since there are no studies conducted to assess the gastric motility in children with abdominal pain, we used gastric motility data obtained for Sri Lankan children with functional abdominal pain [27] to calculate the sample size. At confidence interval of 5 %, power of 80 % and 1:1 ratio between patients and controls, the minimum sample required 32 (16 controls and 16 patients with AM).

#### Statistical analysis

Data were analysed using EpiInfo (EpiInfo version 6.04 (1996), Centres of Disease Control and Prevention, Atlanta, Georgia, USA and World Health Organization, Geneva, Switzerland). The statistical significance of differences of gastric motility parameters between the patient and control groups were assessed using Mann–Whitney *U*-test. Spearman correlation coefficient was used to assess the relationship between gastric emptying parameters and severity of abdominal pain.

## Results

Gastric motility parameters were calculated in 17 children with abdominal migraine and 20 healthy controls. Demographic characteristics of the study sample is shown in Table 2.

**Table 2:** Demographic and family characteristics of children with AM and controls

Variable		AM (*n* = 17)	Controls (*n* = 20)
Gender	Boys	6 (35.3 %)	8 (40.0 %)
*n* (%)	Girls	11 (64.7 %)	12 (60.0 %)
Age distribution	Mean	9.5 years	8,4 years
(years)	SD	3.1 years	3.0 years
	Range	4–15 years	4–14 years
Maternal employment	Leading profession (e.g., doctor, engineer)	1 (5.9 %)	1 (5.0 %)
*n* (%)	Lesser profession (e.g., nurse, teacher)	3 (17.6 %)	4 (20.0 %)
	Skilled non manual (e.g., clerk)	3 (17.6 %)	3 (15.0 %)
	Skilled manual (e.g., mason, carpenter)	1 (5.9 %)	2 (10.0 %)
	Unskilled/unemployed	9 (52.9 %)	10 (50.0 %)
Father’s social class	Leading profession	1 (5.9 %)	1 (5.0 %)
*n* (%)	Lesser profession	5 (29.4 %)	5 (25.0 %)
	Skilled non manual	3 (17.6 %)	4 (20.0 %)
	Skilled manual	5 (29.4 %)	5 (25.0 %)
	Unskilled/unemployed	3 (17.6 %)	5 (25.0 %)
Living area	Urban	7 (41.2 %)	10 (50.0 %)
*n* (%)	Rural	10 (58.8 %)	10 (50.0 %)

### Characteristics of children with AM

Out of 17 children recruited, 12 (60.6 %) had severe abdominal pain and 5 (29.4 %) had very severe abdominal pain. The mean age at onset of symptoms was 8.3 years (SD 3.4 years, median 8.6 years, range 3–14 years), whereas the mean duration of AM was 15.1 months (SD 14.8 months, median 11.5 months, range 2 months to 5 years). The mean duration of pain episodes were 1.6 h (SD 1.3 h, median 1.2 h, range 1–5 h) and the mean frequency of abdominal pain episodes was 20.4 per month (SD 23.7/month, median 11.6/month, range 4–90/month. Some children had several attacks of abdominal pain per day. Mean symptom free period in children with AM varied from 1.8 weeks to 22.3 weeks. Fourteen (82.4 %) children had abdominal pain localized in the peri-umbilical area while 3 (17.6 %) children had pain in a wider area of the abdomen including the umbilical area.

Other intestinal related and extra-intestinal symptoms associated with abdominal pain in children with AM are summarized in Table 3. Symptoms were aggravated by meals in 4 (23.5 %) children, stress in 2 (11.8 %) and physical activity in 1 (5.9 %). None of the children reported any relieving factors.

**Table 3:** Intestinal related and extra-intestinal symptoms in children with abdominal migraine

Symptom	Number	(%)
Headache	11	64.7
Photophobia	8	47.1
Pallor	2	11.8
Dizziness	3	17.6
Lethargy	1	5.9
Joint pain	5	29.4
Nausea	8	47.1
Vomiting	5	29.4
Loss of appetite	5	29.4
Weight loss	5	29.4
Hard stools	2	11.8
Loose stools	5	29.4
Sleep disturbances	1	5.9

Seven (41.2 %) children with AM reported chronic gastrointestinal diseases in first degree relatives while chronic headaches were present in first degree relatives of five (29.4 %) children.

### Gastric motility parameters of children with AM and controls

The results are depicted in Table 4. Children with AM had significantly lower gastric emptying rate, amplitude of antral contractions and antral motility index. Furthermore, their fasting antral area was significantly larger than that of controls.

**Table 4:** Gastric motility parameters in children with abdominal migraine (AM) and controls

	AM *(n = 17)* * Mean (SD)*	Controls *(n = 20)* * Mean (SD)*	*p value* ^*^
Fasting antral area (cm^2^)	1.8 (1.3)	0.6 (1.0)	0.005
Gastric emptying rate (%)	41.6 (13.4)	66.2 (16.5)	<0.0001
Amplitude of antral contractions (%)	57.9 (16.2)	89.0 (10.1)	<0.0001
Frequency of antral contractions (/3 min)	8.8 (0.8)	9.5 (0.8)	0.08
Antral motility index	5.0 (1.5)	8.3 (1.3)	<0.0001

^*^Mann Whitney *U* test

### Correlation between gastric motility parameters and symptom characteristics

The relationship between gastric motility parameters and symptom characteristics are shown in Table 5. Gastric emptying rate had a significant negative correlation with the average duration of pain episodes, while amplitude of antral contractions negatively correlated with scores obtained for severity of symptoms. No significant correlations observed between gastric motility parameters and headache, photophobia, vomiting, nausea and pallor.

**Table 5:** Correlation between gastric motility parameters and symptom characteristics in patients with abdominal migraine

	Scores obtained for severity of abdominal pain	Average duration of a pain episode (min)	Frequency of pain episodes (/month)	Duration of the disease (months)	Age at onset of the disease (years)
Fasting antral area (cm^2^)	0.28	0.30	−0.14	0.08	0.30
Gastric emptying rate (%)	−0.26	−0.58^*^	0.16	−0.04	−0.34
Amplitude of antral contractions (%)	−0.55^*^	−0.43	−0.10	−0.55^*^	0.04
Frequency of antral contractions (/3 min)	−0.33	0.17	0.05	−0.52^*^	0.22
Antral motility index	−0.45	−0.36	−0.17	−0.57^*^	0.07

^*^ *p < 0.05*, Spearman correlation coefficient

### Association between emotional stress and gastric motility

Six (35.3 %) children were exposed to stressful life events during the previous 3 months. When gastric motility parameters between children exposed to stressful events and those not exposed to such events were compared, there was no significant difference (Table 6).

**Table 6:** Gastric motility parameters in children with abdominal migraine according to exposure to stress

	Stressful event positive *Mean (SD)*	Stressful events negative *Mean (SD)*	*p value* ^*^
Fasting antral area (cm^2^)	1.5 (0.5)	1.9 (1.6)	0.8
Gastric emptying rate (%)	43.8 (6.1)	40.2 (16.3)	0.6
Amplitude of antral contractions (%)	50.2 (12.1)	63.1 (17.1)	0.2
Frequency of antral contractions (/3 min)	8.7 (0.5)	8.8 (1.0)	0.7
Antral motility index	4.3 (1.0)	5.5 (1.7)	0.2

^*^Mann Whitney *U* test

## Discussion

The current study describes clinical characteristics of children with AM and their gastric motility abnormalities.

In conformity with an earlier study [14], the majority of children with AM recruited for this study were girls. The mean age of onset of the symptoms of AM (8.3 years) in our study is similar to the observations made in previous studies (7 years) [14]. All children had at least severe abdominal pain lasting for more than 1 h. The average duration of symptoms (1.6 h) was significantly shorter and average frequency of pain episodes (20.4 episodes/month) was significantly higher in our children with AM than previously reported symptoms in adult patients with this condition (41.6 h and 2.0/month respectively) [31]. Although classically pain in AM occurs around the peri-umbilical area, some of our children had pain extending to a wider area of the abdomen. Meal-related symptoms are usually seen in children with FD and IBS. In this sample we found a sizeable proportion (24 %) of children who reported exaggerated pain with a meal. Some children had altered bowel habits as well, although they did not fulfil the criteria for IBS or constipation. Commonest associated symptoms were headache, photophobia and nausea. A previous study conducted in the United Kingdom in children aged 5–15 years has reported anorexia, nausea and pallor as commonest associated symptoms [16].

Despite 0.2 to 23 % of children suffering from AM [7, 11–13, 15–20], the precise mechanism of symptoms remains unknown. Although, the main symptom in children with AM is abdominal pain, they also have symptoms related to dysfunction of the central nervous system such as visual disturbances. Therefore, it is likely that the underlying patho-physiology of AM involves both peripheral and central nervous system dysfunction [32].

Several hypotheses have been investigated to determine the patho-physiology of AM. Factors suggested as underlying mechanisms of pain include IgE-mediated diet induced allergy, gut mucosal immune responses, phenol sulfotransferase enzyme M and P catabolism of catecholamines and monoamines, permeability of the gut mucosal surface and altered relationship between the gut and the central nervous system [33–35]. The enteric nervous system of the gut and the central nervous system arise from the same embryologic tissues. So, it is likely that they have direct effects on each other. Some investigators have proposed that psychological factors such as emotional stress increases central nervous system arousal, which in turn, could lead to dys-regulation of gastrointestinal functions [35].

Gastrointestinal motility abnormalities have been suggested as possible underlying mechanisms for AP-FGIDs. Gastric motility abnormalities have been commonly reported in children with IBS, FD and FAP [25–27, 36–39]. This is the first time gastric motility has been assessed in patients with AM. In this study, we found significantly larger fasting antral area and lower gastric emptying rate and antral motility parameters in a cohort of Sri Lankan children with AM. In addition, we observed significant correlation between some gastric motility parameters and abdominal pain. This is consistent with previous studies conducted in children with FD and FAP, which have reported correlations between abdominal pain and gastrointestinal motility abnormalities [23, 25, 27, 40, 41]. However, we did not observe a similar correlation between headache, nausea, vomiting, photophobia and gastrointestinal motility parameters. All these findings tend to indicate abnormal gastric motility as a potential mechanism that contributes to the patho-physiology of abdominal pain but not to other associated symptoms of AM.

We also assessed the relationship between exposure to stressful life events and gastrointestinal motility in children with AM. We did not observe any significant difference in gastrointestinal motility parameters in children exposed to emotional stress and those not exposed to such events. Previous studies conducted in children with FAP and recurrent abdominal pain also failed to show a difference in gastric motility parameters in children exposed to stress [27, 42]. However, two studies conducted in children with FD and IBS have reported a higher gastric antral area during fasting period and lower gastric emptying rate in those exposed to stressful life events [25, 26].

The exact reason for delay in gastric emptying and abnormal antral motility of AM is not clear. Alterations in brain-gut axis have been commonly suggested as the main pathophysiological mechanism for FGIDs [43]. Psychological factors are proposed to influence gastric functions including sensation, motility, secretion and immunological functions via brain-gut axis [44]. Associated dys-coordination of the antrum and the fundus may partly contribute to the impaired gastric emptying. That in turn leads to stasis of fluid, gases and other contents in the stomach and cause gastric dilatation, which may produce intense pain through stimulated stretch and pain receptors. Hypersensitivity of both central and peripheral neural receptors may have enhanced perception of pain and further increased the pain severity. These physiological phenomena may also contribute to nausea and vomiting. The bi-directional dialogue between brain-gut neurones through the connecting neural and hormonal circuits may have led to the changes in the central nervous system to generate other symptoms such as headache and photophobia. Arousal of autonomic nervous system may give rise to features of sympathetic hyperactivity such as pallor.

In this study we used an ultrasound method to assess gastric motility in children with AM because it is a simple, safe, and non-invasive, and previously used in Sri Lankan children with AP-FGIDs. Ultrasonography has been suggested as a method to detect gastric emptying since 1980 [45]. Thereafter, several techniques were described using ultrasonography [46] and the method described by Bolondi et al*.* [47] laid the foundation for current techniques in measurement of gastric emptying. This method is based on the measurement of the width of gastric antrum before and after a test meal. The gastric antrum is visible in almost all subjects even in patients with obesity [47]. Ultrasound method to assess gastric motility was later described by Hausken et al*.* [48]. The ultrasound methods have been compared with radiological and scintigraphic methods and has proven to be an accurate technique for assessment of gastric emptying [47, 49–53]. The measurements obtained by ultrasound methods have shown a good inter-observer agreement [54]. The technique used in the current study was published by Fujimura and co-workers [55] and subsequently used to assess patients with functional dyspepsia [30]. It was compared with 13-C octonoic breath test and have shown a good agreement [56].

Our study has several strengths. We have investigated children with AM to rule out possible organic diseases causing abdominal pain. Furthermore, significant correlation between motility parameters and symptoms suggest an association between symptoms and physiological correlates. One drawback in our study is inclusion of only a relatively small number of patients. However, AM is not a common disorder and therefore it was not possible to include a very large sample. The other potential limitation is that we included children from a referral centre. One can argue that they may not represent patients in the general population. However, the proposed possible patho-physiological mechanisms are not likely to be altered by selecting the sample from a referral centre. In addition, the investigator who performed the ultrasound measurements was not blinded and was aware that she was scanning a patient with a gastrointestinal problem, even though she did not know the exact diagnosis at the time of scanning. However, the ultrasound measurements done in the current study are objective measurements involving calculations. Therefore we believe that this will reduce the operator bias.

## Conclusions

Gastric emptying rate and antral motility were significantly lower in children and adolescents suffering from abdominal migraine. In addition, we also observed a significant correlation between gastric motility abnormalities and symptoms. Lack of such correlation with extra-intestinal symptoms indicates that gastric motility abnormalities may play a pathophysiological role in the origins of abdominal pain in affected children. More studies are needed to assess the exact relationship between gastrointestinal functions and symptoms in AM.

## Abbreviations

A: Amplitude of antral contractions
AM: Abdominal migraine
AP-FGIDs: Abdominal pain-predominant functional gastrointestinal disorders
F: Frequency of antral contractions
FAP: functional abdominal pain
FD: Functional dyspepsia
FGIDs: Functional gastrointestinal disorders
GE: Gastric emptying rate
IBS: irritable bowel syndrome
KUB: kidney – ureter – bladder
MI: Antral motility index
SD: Standard deviation
